# Patient Satisfaction With the Health Care Services of a Government-Financed Health Protection Scheme in Bangladesh: Cross-Sectional Study

**DOI:** 10.2196/49815

**Published:** 2024-04-24

**Authors:** Md Zahid Hasan, Md Golam Rabbani, Orin Akter, Gazi Golam Mehdi, Mohammad Wahid Ahmed, Sayem Ahmed, Mahbub Elahi Chowdhury

**Affiliations:** 1 Health Systems and Population Studies Division icddr,b Dhaka Bangladesh; 2 Leeds Institute of Health Sciences University of Leeds Leeds United Kingdom; 3 Health Economics and Health Technology Assessment School of Health & Wellbeing University of Glasgow Glasgow United Kingdom

**Keywords:** Shasthyo Surokhsha Karmasuchi, health care services, health care utilization, satisfaction, below poverty line, Bangladesh, patient satisfaction, physician behavior

## Abstract

**Background:**

Since 2016, the government of Bangladesh has been piloting a health protection scheme known as Shasthyo Surokhsha Karmasuchi (SSK), which specifically targets households living below the poverty line. This noncontributory scheme provides enrolled households access to inpatient health care services for 78 disease groups. Understanding patients’ experiences with health care utilization from the pilot SSK scheme is important for enhancing the quality of health care service delivery during the national-level scale-up of the scheme.

**Objective:**

We aimed to evaluate patient satisfaction with the health care services provided under the pilot health protection scheme in Bangladesh.

**Methods:**

A cross-sectional survey was conducted with the users of the SSK scheme from August to November 2019. Patients who had spent a minimum of 2 nights at health care facilities were selected for face-to-face exit interviews. During these interviews, we collected information on patients’ socioeconomic characteristics, care-seeking experiences, and level of satisfaction with various aspects of health care service delivery. To measure satisfaction, we employed a 5-point Likert scale (very satisfied, 5; satisfied, 4; neither satisfied nor dissatisfied, 3; dissatisfied, 2; very dissatisfied, 1). Descriptive statistics, statistical inferential tests (*t*-test and 1-way ANOVA), and linear regression analyses were performed.

**Results:**

We found that 55.1% (241/438) of users were either very satisfied or satisfied with the health care services of the SSK scheme. The most satisfactory indicators were related to privacy maintained during diagnostic tests (mean 3.91, SD 0.64), physicians’ behaviors (mean 3.86, SD 0.77), services provided at the registration booth (mean 3.86, SD 0.62), confidentiality maintained regarding diseases (mean 3.78, SD 0.72), and nurses’ behaviors (mean 3.60, SD 0.83). Poor satisfaction was identified in the interaction of patients with providers about illness-related information (mean 2.14, SD 1.40), availability of drinking water (mean 1.46, SD 0.76), cleanliness of toilets (mean 2.85, SD 1.04), and cleanliness of the waiting room (mean 2.92, SD 1.09). Patient satisfaction significantly decreased by 0.20 points for registration times of 16-30 minutes and by 0.32 points for registration times of >30 minutes compared with registration times of ≤15 minutes. Similarly, patient satisfaction significantly decreased with an increase in the waiting time to obtain services. However, the satisfaction of users significantly increased if they received a complete course of medicines and all prescribed diagnostic services.

**Conclusions:**

More than half of the users were satisfied with the services provided under the SSK scheme. However, there is scope for improving user satisfaction. To improve the satisfaction level, the SSK scheme implementation authorities should pay attention to reducing the registration time and waiting time to obtain services and improving the availability of drugs and prescribed diagnostic services. The authorities should also ensure the supply of drinking water and enhance the cleanliness of the facility.

## Introduction

Globally, more than half of the population encounters difficulties in accessing essential health care services, with the majority residing in low- and middle-income countries (LMICs) [1]. These nations experience substantial challenges in financing health care [2-5]. Consequently, health care financing in these countries heavily relies on out-of-pocket spending by households, leading to increased financial distress on families during their illness [2,3,6]. In many instances, the most affected are those in poverty, and they lack access to health care services when they are unwell [7]. Similar to other LMICs, out-of-pocket spending for health care in Bangladesh is notably high. Recent evidence indicates that 68.5% of the total health care expenditure is shouldered by households through out-of-pocket payments [8]. Another recent study reported that such high out-of-pocket payments resulted in 24.6% of households experiencing catastrophic health expenditure when estimated using the 10% threshold of the budget share method. Furthermore, in 2016, over 8.5 million people were pushed into poverty due to health care expenses [9]. Moreover, the incidence of catastrophic health expenditure is more concentrated among the poorest households (16.5%) compared to the richest (9.2%) [10]. To reduce the burden of health care among the population and progress toward universal health coverage, the Government of Bangladesh has developed the Health Care Financing Strategy 2012–2032, intending to provide financial protection for health care to all citizens by 2032 [11]. As a component of this strategy, the Health Economics Unit of the Ministry of Health and Family Welfare of the Government of Bangladesh has been implementing a social health protection scheme known as “Shasthyo Surokhsha Karmasuchi (SSK)” since 2016. Although there is a comprehensive plan to cover the entire population of the country within the financing scheme, the current implementation is limited to a noncontributory scheme focusing on the below-poverty-line population. The scheme is being piloted in 3 subdistricts: Kalihati, Madhupur, and Ghatail under Tangail District. The scheme has enrolled almost 1,00,000 households that have access to inpatient health care services from Upazila Health Complexes (UzHCs) of the respective Upazilas (subdistricts) and district hospitals. Participation in the scheme is mandatory for households identified as being below the poverty line, and their enrollment is noncontributory, meaning that these enrolled households are not required to pay any fees for services. Notably, the scheme does not offer purchasing services to the above-poverty-line population. The government established a pool fund, allocating BDT 1000 (US$12) per household per year as a premium (BDT 84.5 = US$ 1, August 2019, Bangladesh Bank). This measure ensures access to inpatient health care services for the enrolled below-poverty-line households, covering 78 different disease groups. The annual coverage limit for each household is BDT 50,000 (US $592). Under the scheme, inpatient health care is delivered through UzHCs, serving as the first access point for the insured beneficiaries to receive health care services. Through a structured referral system, the beneficiaries can also access services at the Tangail District Hospital. The scheme ensures that insured patients receive free diagnostic services and medicines through hospitals, contracted diagnostic centers, and pharmacies. The SSK management authority, scheme operator, hospitals, contracted diagnostic centers, and pharmacies play crucial roles in the implementation of the scheme [12].

Although the scheme provides free inpatient care services to the member households, the health care utilization under the SSK scheme is notably low. A study revealed that less than half of the beneficiary households used health care services under the SSK scheme [13]. Several factors may contribute to this low utilization rate. For instance, quality of care might be a significant factor among the various important determinants of health service utilization. Quality of service is recognized as one of the key components in achieving universal health coverage by its definition [14]. Traditionally, the quality of health care services was primarily assessed based on professional practice standards. However, in the recent decades, patients’ perceptions of health care have emerged as an important indicator for evaluating the quality of health care services. Various studies have demonstrated that health service utilization is closely linked with users’ perceptions of the quality of health care provided [15-17]. Consequently, patient satisfaction is considered as an important aspect of performance improvement of the delivered health care services, alongside clinical effectiveness. It is a multidimensional aspect where patients’ perceptions and attitudes shape their overall health care–seeking experience [18,19]. Several factors, including registration time and process, waiting time to obtain health care services, interpersonal communication, and availability of basic amenities within health care facilities, can influence patient satisfaction with health care services [20-23]. Increased utilization and satisfaction of any insurance scheme are associated with improved quality of health care services. However, the literature provides mixed evidence. For example, a study in India found no significant difference in satisfaction levels between insured and uninsured hospitalized patients [24]. Conversely, regarding the overall quality of care provided under the National Health Insurance Scheme of Ghana, a significant portion of insured patients reported higher satisfaction compared with uninsured patients [25]. Evidence from Nigeria indicated that most patients were satisfied with the service delivery of their national health insurance scheme [26-28]. In Ethiopia, a study revealed that approximately 55% of enrollees were satisfied with the community-based health insurance scheme [29], whereas another study from the same country indicated that over 90% of households were satisfied with the community-based health insurance scheme [30]. Different Vietnamese studies have reported poor satisfaction among beneficiaries regarding service coverage and quality of care under national health insurance [31,32]. A recent study conducted on a self-financed health insurance scheme in Bangladesh showed that, overall, members of the scheme were satisfied with the health care services; however, their satisfaction levels could be improved in several aspects of health care service delivery [33].

Despite the pilot implementation of the SSK scheme since 2016 and its low utilization, no research has been conducted on service users’ experiences and levels of satisfaction with the scheme. Gaining a better understanding of beneficiaries’ experiences and levels of satisfaction with the health care service provided under the pilot SSK scheme is crucial. This insight can help identify the gaps in the quality of health care services provided. Such evidence will be useful for the key stakeholders of the health protection scheme, allowing them to make necessary changes in the service delivery process and related aspects to enhance the quality of health care services provided under the scheme. As a result, this study was conducted to address 2 central research questions: (1) What is the level of satisfaction among the beneficiaries of the SSK scheme? and (2) What are the factors influencing their satisfaction level? In addressing these research questions, this study aimed to assess patients’ levels of satisfaction with the services offered by the SSK scheme in Bangladesh.

## Methods

### Study Design

A cross-sectional exit patient survey was designed to gain insights into the experiences of insured patients with various aspects of the service delivery process and the quality of services provided under the pilot SSK scheme. Every second patient who had been admitted for at least 2 nights at a scheme-designated facility was selected and interviewed at the time of discharge.

### Study Setting and Sample

The study was conducted in the UzHCs of Kalihati, Madhupur, and Ghatail Upazilas (subdistricts), and Tangail District Hospital of Tangail District. Insured inpatients were interviewed after discharge from the health care facilities. The survey of the respondents took place between August 18 and November 16, 2019, on working days, from Saturday to Thursday. Every second discharged inpatient from the male and female wards was interviewed. To ensure the quality of the data, a maximum of 4 patients were interviewed each day at an SSK hospital. A total of 438 discharged inpatients aged 18 years or older were interviewed from 3 UzHCs (Kalihati, n=128; Madhupur, n=176; and Ghatail, n=134) and Tangail District Hospital (n=88).

### Data Collection Process

A semistructured questionnaire was designed and pretested before data collection. Face-to-face interviews were conducted with the insured patients and, in certain cases, with attendants of patients at the time of discharge. An attendant was considered as a respondent when the patient was not involved with the various dimensions of the service delivery process during the inpatient episode owing to the physical condition.

The questionnaire covered demographic and socioeconomic details of the respondents and households, health care utilization, and various dimensions of satisfaction related to the SSK scheme. These dimensions included the registration process at the SSK booth, the dignity of patients during treatment, clear communication with health care providers, privacy during treatment, the quality of basic amenities, the availability of drugs and supplies, and the availability of prescribed diagnostic services.

Four experienced research assistants were employed for patient recruitment and conducting the interviews. Prior to the interviews, written informed consent was obtained from all participants, and their participation was entirely voluntary. Completed interviews were cross-checked among the interviewers and further reviewed by the supervisor to ensure data quality and to address any associated issues, if needed, during the data collection.

### Study Variables

We collected information on various background characteristics of the patients, including age, sex, education level, current employment status, current marital status, and family size. For measuring satisfaction levels, we considered several dimensions of health care delivery under the SSK scheme.

The first dimension was hospitalization-related factors. It included self-reported illnesses and length of stay. Self-reported illnesses were categorized into 3 groups: communicable, noncommunicable, and others (ie, obstetrics and injury). Communicable diseases encompass illnesses caused by viruses or bacteria that spread through contact, bodily fluids, blood products, insect bites, or the air. Noncommunicable diseases, on the other hand, are those that do not transmit between individuals and often necessitate long-term treatment.

The second dimension was service utilization–related aspects. It included waiting time for registration, waiting time to obtain health care services, behavior of health care providers (including physicians, nurses, and other staff, such as ward boys and cleaners), interaction of health care providers with patients, privacy during diagnostic services, and confidentiality of the health care provided.

The third dimension was facility environment and basic amenity–related factors. It included cleanliness of health facilities, waiting rooms, and toilets, and availability of drinking water.

The satisfaction measurement items demonstrated a satisfactory level of internal consistency, as indicated by an overall Cronbach α coefficient of 0.77 out of 1.0 [34].

### Satisfaction Measurements

Patient satisfaction was measured with a collective outcome of 14 different items. The selection of items for measurements was devised based on a literature review of patient satisfaction with the insurance scheme as well as previous systematic reviews [26,27,33,35-40]. The existing literature has examined various aspects of health service delivery from the patients’ viewpoints, encompassing domains such as patient-provider interactions, the physical environment, and internal management processes. We selected items that revolved around these domains as they encompassed the most influential satisfaction constructs. The 14 items are presented in Textbox 1.

Each considered item was rated on a 5-point Likert scale (very satisfied, 5; satisfied, 4; neither satisfied nor dissatisfied, 3; dissatisfied, 2; very dissatisfied, 1). The total satisfaction score of respondents for all items ranged from a minimum of 14 to a maximum of 70. Furthermore, we included an item in the questionnaire to assess the overall satisfaction (on a scale of 5) with the services at the SSK facility.

**Items for patient satisfaction.**

**Satisfaction items**

- How will you rate the behavior of the authority of Shasthyo Surokhsha Karmasuchi (SSK) at the registration booth?
- What is your opinion about the time taken for completing registration?
- What is your opinion about the waiting time before consultation with the service provider?
- How will you rate the behavior of the service provider during your treatment at this hospital?
- How will you rate the behavior of nurses during your treatment at this hospital?
- How will you rate the behavior of the aya/ward boy during your treatment at this hospital?
- How will you rate the interaction with the service provider about your illness and treatment?
- How will you rate the doctor’s attitude toward listening to your problems?
- How will you rate the privacy maintained during diagnostic tests?
- What is your opinion about the privacy maintained during consultation?
- What is your opinion about the cleanliness of this hospital?
- How will you rate the cleanliness of the waiting room of this hospital?
- How will you rate the cleanliness of the toilets of this hospital?
- What is your opinion regarding the availability of drinking water in the hospital?

### Statistical Analysis

We analyzed the data using Stata version 16 (StataCorp) [41]. We performed both descriptive analysis and statistical inferential tests to measure the association between dependent and independent variables. In the descriptive analysis, background characteristics of the study participants and health care facility utilization–related characteristics were presented in terms of frequency (n) and percentage (%) with 95% CIs. Moreover, we performed a *t*-test for variables with 2 categories and 1-way ANOVA for variables with more than 2 categories to test the significant differences in average satisfaction levels across the demographic and socioeconomic characteristics related to the SSK scheme.

To identify factors associated with patients’ average scores for satisfaction with the services under the SSK scheme, a linear regression analysis was performed. We estimated the satisfaction level for each patient by taking the average of the reported satisfaction levels in the 14 items. In the univariate unadjusted regression model, the dependent variable was the mean satisfaction score and the independent variables were age, gender, education, employment status, marital status, family size, self-reported illness, length of hospitalization, registration time, waiting time to obtain services, status of receiving drugs and supplies, and status of receiving diagnostic services. However, in the multivariate regression model, we included independent variables that had a significant association with the satisfaction score (ie, *P* values ≤.05) in the univariate regression models. We considered *P* values of <.05 as statistically significant in our analysis.

### Ethics Approval

This study was approved by the Research Review Committee and Ethical Review Committee of the icddr,b (protocol#: PR-17047). Participants in the study were recruited and interviewed after obtaining written informed consent, and their participation was voluntary.

## Results

### Descriptive Statistics

A total of 438 patients aged 18 years and above were interviewed at the studied facilities (Table 1), and 60.1% (263/438) of the patients were female. According to education level, 60.9% (267/438) of patients had no education, whereas 24.2% (106/438) and 14.8% (65/438) had primary and secondary levels of education, respectively. Moreover, 67.8% (297/438) of patients were not involved with income generation. In terms of marital status, 83.3% (365/438) of patients were married. Moreover, 54.1% (237/438) were from a household consisting of more than 4 members.

**Table 1:** Characteristics of participants and their hospital service utilization.

Variable					Value (N=438), n (%)
**Participant variable**					
	**Age (years)**				
		18-44	158 (36.1)		
		45-64	202 (46.1)		
		>64	78 (17.8)		
	**Sex**				
		Male	175 (39.9)		
		Female	263 (60.1)		
	**Education level**				
		No education	267 (61.0)		
		Primary	106 (24.2)		
		Secondary or higher	65 (14.8)		
	**Employment status**				
		Employed	141 (32.2)		
		Unemployed	197 (44.0)		
		Retired or student	100 (22.8)		
	**Marital status**				
		Unmarried	11 (2.5)		
		Married	365 (83.3)		
		Widowed, divorced, or separated	62 (14.2)		
	**Family size**				
		≤4 members	201 (45.9)		
		>4 members	237 (54.1)		
	**Self-reported illness**				
		Communicable	135 (30.8)		
		Noncommunicable	274 (62.6)		
		Others (ie, obstetrics and injury)	29 (6.6)		
**Hospital service utilization variable**					
	**Length of hospitalization (days)**				
		2	151 (34.5)		
		3-4	208 (47.5)		
		>4	79 (18.0)		
	**Registration time (min)**				
		≤15	290 (66.2)		
		16-30	91 (20.8)		
		>30	57 (13.0)		
	**Waiting time to get services (min)**				
		≤15	258 (58.9)		
		16-30	84 (19.2)		
		>30	96 (21.9)		
	**Status of getting drugs and supplies**				
		Partially received	91 (20.8)		
		All received	347 (79.2)		
	**Status of getting laboratory services**				
		Not prescribed	74 (16.9)		
		Partially received	38 (8.7)		
		All received	326 (74.4)		

According to self-reported diseases, 62.6% (274/438) of patients reported the reason for hospitalization as noncommunicable disease, 30.8% (135/438) reported the reason as communicable disease, and 7.0% (29/438) reported the reason as other health problems (ie, obstetrics and injury). Regarding the length of hospitalization, 47.5% (208/438) of patients were admitted for 3-4 days, 34.5% (151/438) were admitted for 2 days, and 18.0% (79/438) were admitted for more than 4 days. Among the respondents, 66.2% (290/438) mentioned that they had completed their registration process within 15 minutes, and 58.9% (258/438) waited for 15 minutes or less to get services. The majority of patients (347/438, 79.2%) received all prescribed medicines and supplies free from the SSK pharmacy. Regarding laboratory services, 74.4% (326/438) of patients reported that they received diagnostic services as prescribed. More details of the descriptive statistics are shown in Table 1.

### Level of Satisfaction by Different Items

Patient satisfaction with the items considered while using the SSK scheme is shown in Table 2. A total of 14 satisfaction items were used to examine patient satisfaction. The highest average score on satisfaction was related to “privacy maintained during diagnostic tests” (mean 3.91, SD 0.64), followed by “physicians’ behaviors” (mean 3.86, SD 0.77), “services at the SSK registration booth” (mean 3.86, SD 0.62), “confidentiality maintained about diseases” (mean 3.78, SD 0.72), and “services from nurses” (mean 3.6, SD 0.83). Among service-related items, a lower level of satisfaction was reported for the interaction of service providers with patients (mean 2.14, SD 1.4). Among the items in the environment and basic amenities domain, comparatively higher satisfaction was found for the cleanliness of the health facility (mean 3.43, SD 0.76), followed by the cleanliness of the waiting room (mean 2.92, SD 1.09) and toilets (mean 2.85, SD 1.04). The lowest level of satisfaction was reported for the availability of drinking water (mean 1.46, SD 0.76).

**Table 2:** Patient satisfaction with health care services at Shasthyo Surokhsha Karmasuchi facilities by different items (N=438).

Item	Very satisfied, n (%)	Satisfied, n (%)	Neutral, n (%)	Dissatisfied, n (%)	Very dissatisfied, n (%)	Overall score, mean (SD)
1. Services at the SSK^a^ registration booth (reception)	30 (6.9)	338 (77.2)	51 (11.6)	15 (3.4)	4 (0.9)	3.86 (0.62)
2. Registration time	53 (12.1)	191 (43.6)	93 (21.2)	48 (11.0)	53 (12.1)	3.33 (1.19)
3. Waiting time to get health care services	74 (16.9)	146 (33.3)	78 (17.8)	61 (13.9)	79 (18.0)	3.17 (1.36)
4. Physicians’ behaviors	52 (11.9)	314 (71.7)	42 (9.6)	20 (4.6)	10 (2.3)	3.86 (0.77)
5. Nurses’ behaviors	29 (6.6)	265 (60.5)	94 (21.5)	40 (9.1)	10 (2.3)	3.60 (0.83)
6. Other staff behaviors	12 (2.7)	249 (56.9)	117 (26.7)	44 (10.1)	16 (3.7)	3.45 (0.85)
7. Interaction of health care providers with patients regarding illness	30 (6.9)	83 (19.0)	32 (7.3)	65 (14.8)	228 (52.1)	2.14 (1.40)
8. Empathy of health care providers	38 (8.7)	173 (40.0)	126 (28.8)	69 (15.8)	32 (7.3)	3.27 (1.06)
9. Privacy during diagnostics among patients who got diagnostic tests	47 (12.9)	250 (68.7)	54 (14.8)	13 (3.6)	0 (0.0)	3.91 (0.64)
10. Confidentiality of diseases	40 (9.1)	292 (66.7)	83 (19.0)	17 (3.9)	6 (1.4)	3.78 (0.72)
11. Cleanliness of the health facility	10 (2.3)	226 (51.6)	151 (34.5)	45 (10.3)	6 (1.4)	3.43 (0.76)
12. Cleanliness of the waiting room	4 (0.9)	163 (37.2)	133 (30.4)	71 (16.2)	67 (15.3)	2.92 (1.09)
13. Cleanliness of toilets	8 (1.8)	132 (30.1)	134 (30.6)	113 (25.8)	51 (11.6)	2.85 (1.04)
14. Availability of drinking water	2 (0.5)	16 (3.7)	11 (2.5)	125 (28.5)	284 (64.8)	1.46 (0.76)

^a^SSK: Shasthyo Surokhsha Karmasuchi.

### Overall Patient Satisfaction With Health Care Services at SSK Facilities

Considering the response to the overall patient satisfaction with the services at SSK facilities, 8.5% (37/438) reported being very satisfied and 46.6% (204/438) reported being satisfied with the services received under the SSK scheme. On the other hand, 31.3% (137/438) of respondents reported feeling neither satisfied nor dissatisfied. Moreover, 8.9% (39/438) were dissatisfied and 4.8% (21/438) were very dissatisfied (Multimedia Appendix 1).

### Patient Satisfaction by Socioeconomic and Hospital Service Utilization Characteristics

Patient satisfaction levels significantly varied across different groups of age, sex, marital status, illness type, registration time, waiting time, status of receiving drugs, and status of getting diagnostic tests (Table 3). Patients aged between 45 and 64 years were comparatively more satisfied (mean 3.28, 95% CI 3.21-3.34) with services under the SSK scheme, and the difference in the satisfaction level across the age groups was statistically significant (*P*<.001). Male patients were significantly (*P*=.01) more satisfied (mean 3.24, 95% CI 3.17-3.31) than female patients. Married and widowed, divorced, or separated individuals were more satisfied than unmarried individuals, and the difference was statistically significant (*P*<.001). However, there was no significant difference in satisfaction by education level, employment status, or household size.

Patients with noncommunicable diseases had a higher satisfaction level (mean 3.22, 95% CI 3.17-3.28) than patients with other illnesses, and the difference in the satisfaction level was statistically significant (*P*=.008). Satisfaction scores decreased with increases in the length of hospitalization, registration time, and waiting time. The satisfaction level was significantly (*P*=.006) higher among patients who received all prescribed drugs from the scheme (mean 3.20, 95% CI 3.15-3.26). Similarly, the satisfaction level was higher among patients who received all prescribed diagnostic or laboratory services compared with other groups (mean 3.22, 95% CI 3.17-3.27), and the difference in the satisfaction level across the groups was statistically significant (*P*<.001).

**Table 3:** Satisfaction scores by patient and service characteristics.

Variable			Score, mean (95% CI)		*P* value
**Age (years)**					<.001^a^
	18-44	3.03 (2.95-3.10)			
	45-64	3.28 (3.21-3.34)			
	>64	3.18 (3.08-3.28)			
**Sex**					.01^b^
	Male	3.24 (3.17-3.31)			
	Female	3.12 (3.06-3.18)			
**Education level**					.21^a^
	No education	3.19 (3.13-3.24)			
	Primary	3.17 (3.07-3.28)			
	Secondary or higher	3.08 (2.96-3.20)			
**Employment status**					.47^a^
	Employed	3.22 (3.14-3.30)			
	Unemployed	3.16 (3.09-3.23)			
	Retired and student	3.12 (3.03-3.21)			
**Marital status**					<.001^a^
	Unmarried	2.65 (2.42-2.88)			
	Married	3.19 (3.14-3.24)			
	Widowed, divorced, or separated	3.12 (2.99-3.24)			
**Family size**					.21^b^
	≤4	3.20 (3.14-3.26)			
	>4	3.14 (3.08-3.21)			
**Self-reported illness**					.008^a^
	Communicable	3.07 (2.98-3.16)			
	Noncommunicable	3.22 (3.17-3.28)			
	Others (ie, obstetrics and injury)	3.10 (2.93-3.27)			
**Length of hospitalization (days)**					.13^a^
	2	3.12 (3.03-3.21)			
	3-4	3.17 (3.11-3.23)			
	>4	3.26 (3.16-3.35)			
**Registration time (min)**					<.001^a^
	≤15	3.25 (3.20-3.30)			
	16-30	3.05 (2.95-3.16)			
	>30	2.93 (2.80-3.07)			
**Waiting time to get services (min)**					<.001^a^
	≤15	3.31 (3.25-3.37)			
	16-30	3.01 (2.91-3.12)			
	>30	2.92 (2.83-3.01)			
**Status of getting drugs and supplies**					.006^b^
	Partially received	3.04 (2.96-3.13)			
	All received	3.20 (3.15-3.26)			
**Status of getting laboratory services**					<.001^a^
	Not prescribed	2.94 (2.81-3.06)			
	Partially received	3.16 (3.02-3.30)			
	All received	3.22 (3.17-3.27)			
Total score			3.17 (3.12-3.21)		

^a^One-way ANOVA.

^b^*t*-test.

### Determinants of Patient Satisfaction With Services Provided Under the SSK Scheme

Our analysis demonstrated noteworthy associations between satisfaction scores and various factors (Table 4). The satisfaction score was significantly higher by 0.13 points in patients aged between 45 and 64 years than in patients aged between 18 and 44 years. Additionally, the satisfaction score was significantly higher by 0.34 points in married patients than in unmarried patients. Moreover, the satisfaction score was significantly higher by 0.15 points in patients seeking care for noncommunicable diseases than in patients seeking care for communicable diseases. We found a significant negative association of the satisfaction score with extended registration and waiting time for obtaining services. Conversely, a positive association was observed with the status of receiving all drugs, supplies, and diagnostic services. The satisfaction score was significantly lower by 0.18 points in patients with a registration time of 16-30 minutes and by 0.33 points in patients with a registration time of >30 minutes than in patients with a registration time of ≤15 minutes. Similarly, the satisfaction score was significantly lower by 0.30 points in patients who waited for 16-30 minutes to obtain services and by 0.36 points in patients who waited for >30 minutes to obtain services than in patients who waited for ≤15 minutes to obtain services. Moreover, the satisfaction score was significantly higher by 0.13 points in patients who received the complete course of prescribed medicines from the SSK pharmacy than in patients who received partial medicines and supplies. Likewise, the satisfaction score was significantly higher by 0.26 points in patients who received partial diagnostic services and by 0.28 points in patients who received full diagnostic services than in patients who were not prescribed diagnostic services.

**Table 4:** Determinants of patient satisfaction with services under the Shasthyo Surokhsha Karmasuchi scheme.

Variable^a^			Unadjusted coefficient^b^, value (95% CI)		*P* value		Adjusted coefficient^b^, value (95% CI)		*P* value
**Age**									
	18-44 (reference)	N/A^c^				N/A			
	45-64	0.25 (0.15 to 0.35)		<.001		0.13 (0.03 to 0.22)		.009	
	>64	0.15 (0.02 to 0.28)		.02		0.03 (−0.10 to 0.16)		.61	
**Sex**									
	Female (reference)	N/A				N/A			
	Male	0.12 (0.02 to 0.21)		.01		0.09 (0.00 to 0.18)		.043	
**Education level**									
	No education (reference)	N/A				N/A			
	Primary	−0.02 (−0.13 to 0.09)		.74		N/A			
	Secondary or higher	−0.10 (−0.24 to 0.02)		.11		N/A			
**Employment status**									
	Employed (reference)	N/A				N/A			
	Unemployed	−0.06 (−0.17 to 0.05)		.26		N/A			
	Retired or student	−0.10 (−0.22 to 0.03)		.13		N/A			
**Marital status**									
	Unmarried (reference)	N/A				N/A			
	Married	0.54 (0.25 to 0.83)		<.001		0.34 (0.08 to 0.61)		.01	
	Widowed, divorced, or separated	0.47 (0.16 to 0.78)		.003		0.26 (−0.03 to 0.55)		.08	
**Family size**									
	≤4 (reference)	N/A				N/A			
	>4	0.06 (−0.03 to 0.15)		.21		N/A			
**Self-reported illness**									
	Communicable (reference)	N/A				N/A			
	Noncommunicable	0.15 (0.05 to 0.25)		.003		0.10 (0.01 to 0.19)		.03	
	Others (ie, obstetrics and injury)	0.03 (−0.16 to 0.23)		.74		0.01 (−0.16 to 0.18)		.91	
**Length of hospitalization (days)**									
	2 (reference)	N/A				N/A			
	3-4	0.05 (−0.05 to 0.15)		.33		0.02 (−0.07 to 0.11)		.63	
	>4	0.14 (0.00 to 0.27)		.045		0.06 (−0.06 to 0.18)		.32	
**Registration time (min)**									
	≤15 (reference)	N/A				N/A			
	16-30	−0.20 (−0.31 to −0.08)		<.001		−0.18 (−0.28 to −0.09)		<.001	
	>30	−0.32 (−0.46 to −0.19)		<.001		−0.33 (−0.45 to −0.21)		<.001	
**Waiting time to get services (min)**									
	≤15 (reference)	N/A				N/A			
	16-30	−0.30 (−0.41 to −0.18)		<.001		−0.30 (−0.40 to −0.20)		<.001	
	>30	−0.39 (−0.49 to −0.28)		<.001		−0.36 (−0.46 to −0.26)		<.001	
**Status of getting drugs and supplies**									
	Partially received (reference)	N/A				N/A			
	All received	0.16 (0.05 to 0.27)		.01		0.13 (0.04 to 0.23)		.008	
**Status of getting laboratory services**									
	Not prescribed (reference)	N/A				N/A			
	Partially received	0.22 (0.04 to 0.41)		.02		0.26 (0.09 to 0.43)		.002	
	All received	0.29 (0.17 to 0.41)		<.001		0.28 (0.17 to 0.39)		<.001	

^a^The dependent variable is the average satisfaction score of 14 items.

^b^The number of observations was 438, R-square value was 0.319, and adjusted R-square value was 0.293.

^c^N/A: not applicable.

## Discussion

### Principal Results and Comparison With Prior Work

We found that 55.1% (241/438) of patients were either very satisfied or satisfied with the services provided by the SSK health protection scheme. The mean satisfaction score was 3.17 out of 5, which means that, on average, the satisfaction level among the patients was slightly above the level of neither satisfied nor dissatisfied. Regarding the 14 considered items for measuring satisfaction, most of the patients were either very satisfied or satisfied with services at the SSK center (368/438, 84.0%), physicians’ behaviors (366/438, 83.6%), and privacy maintained during diagnostic services (297/364, 81.6%). On the other hand, majority of the patients were either very dissatisfied or dissatisfied with the availability of drinking water (409/438, 93.4%) and interaction with health care providers (293/438, 66.9%) regarding the illness. In multiple regression analysis, we found that receiving prescribed drugs and diagnostic services, the waiting time for registration, and the waiting time for getting treatment were the strongest predictors of patient satisfaction.

Health financing schemes are becoming popular to maintain and improve the health of the population in LMICs [2,6,42]. The SSK health protection scheme has been introduced to increase the access of the poor population to inpatient health care services and ensure financial protection against expenditure to alleviate poverty or extreme poverty induced by out-of-pocket payments for health care in Bangladesh. Although several studies have been conducted on patient satisfaction with health care utilization in different settings in Bangladesh [33,43-46], patient satisfaction with services under the SSK health protection scheme has not been studied thus far. The mean satisfaction score in our study was higher than that in a study conducted to assess satisfaction with the service quality of UzHCs among the uninsured population (3.17 vs 2.75) [44]. The SSK scheme provides health care to members through selected UzHCs; however, compared with nonmembers, insured patients are supposed to receive all prescribed medicines and diagnostic services from private providers contracted by the scheme [13]. The situation is different for other UzHCs where the SSK scheme is not being implemented. The availability of medicines and diagnostic services under the SSK scheme might have increased the satisfaction level among the insured patients.

Our study showed that patient satisfaction was the highest regarding the privacy and confidentiality maintained by providers during diagnostic tests and the patients’ diseases. The finding is similar to that in a study conducted in Bangladesh [33] among the beneficiaries of a community-based health insurance scheme. Another study conducted among adult patients at a general hospital in Ethiopia also reported that patient privacy and confidentiality maintained by health care providers were significantly associated with higher satisfaction levels [47]. Our study found that patients were satisfied with providers’ behaviors, particularly physicians’ and nurses’ behaviors, which influenced the overall level of patient satisfaction. Although not directly comparable, the proportion of patients satisfied with the behavior of providers was higher than the proportion reported in a study conducted in rural Bangladesh (84% vs 69%) [45]. Previous studies have also reported that the behavior of health care providers toward patients is directly connected with patient satisfaction [33,43,48].

Regarding interactions with health care providers, our study found that two-thirds of patients were not satisfied. This might be the result of patients not knowing about their illnesses from physicians during their treatment episodes. It is evident from the literature that patients’ satisfaction levels are influenced by healthy interpersonal communication with health care providers as this maintains a better physician-patient relationship [43]. A previous study conducted in Bangladesh showed that more than half of the surveyed patients could not ask questions to their providers about their illness [49]. However, as all patients in our study were inpatients and stayed at the facility for at least 2 days, it is unlikely that patients could not ask their providers about their illness.

Patient experiences with the cleanliness of health facilities and toilets and the availability of drinking water were not positive. Previous studies revealed that the health facility environment and cleanliness were crucial aspects of patient satisfaction [33,50-52]. Moreover, evidence indicates that since environmental contamination is directly connected with nosocomial infection, the physical environment can lead to the dissatisfaction of patients at health facilities instead of increasing satisfaction [33,50-52].

We found that patient age was significantly associated with the level of satisfaction. Another study conducted in Bangladesh [44] reported significant variation in the average satisfaction score across patient age, which is similar to our findings. Two other studies conducted among beneficiaries of health insurance schemes also reported similar findings that age was significantly associated with the level of satisfaction [30,53]. Lower waiting times for registration and health care were significantly associated with patient satisfaction. The findings are consistent with the findings that prolonged waiting times for registration and services are associated with lower client satisfaction [54,55]. Patients who received care for noncommunicable diseases were significantly more satisfied than patients having communicable diseases. This might be because people having noncommunicable diseases require regular medications, which are common and available through the contracted pharmacy. Such availability of medicines might have increased patient satisfaction. Similarly, SSK beneficiaries who received all prescribed medicines and diagnostic services were significantly more satisfied. According to the benefits package of the SSK scheme, patients should receive all prescribed medicines and diagnostic services for 78 disease groups. However, 20.8% (91/438) of patients reported that they received partial medicines and 8.7% (38/438) reported that they received partial diagnostic services. It might have happened that some of the prescribed medicines or diagnostic tests were not correlated with the 78 disease categories and therefore were not provided under the scheme. However, evidence indicates that medicines and diagnostic tests are associated with higher out-of-pocket expenditure and lead to falling into poverty [9,56,57]. Scheme beneficiaries are provided free essential medicines and free diagnostic services, and they have a low chance of incurring treatment costs and experience low risks of catastrophic health expenditure, impoverishment, and further impoverishment [9], thus increasing their satisfaction with the services under the scheme. However, other variables, such as education level, employment status, family size, and length of hospitalization, were not significantly associated with satisfaction levels. This might be because the SSK scheme targets the below-poverty-line population having relatively similar socioeconomic characteristics; thus, their perceptions of satisfaction do not vary across these factors. These findings are consistent with the findings of other studies conducted in India [24] and Turkey [37].

This is the first study to explore patient satisfaction with the pilot SSK scheme in Bangladesh. Furthermore, we included patients from all 4 facilities under the SSK scheme rather than selecting them purposively. The findings of this study will help SSK implementation authorities to understand the patient experience of the service delivery process and the quality of health care provided under the SSK scheme.

### Limitations

The design of this study was observational in nature, which did not allow us to establish any causal inference with satisfaction and other characteristics under the SSK scheme without a control group. The study only focused on the point of view of the beneficiaries, and we did not explore the providers’ views in this context. The survey collected self-reported satisfaction information from patients, which is highly susceptible to social desirability bias as patients might give responses that please health care providers instead of truly reflecting their satisfaction. However, we interviewed patients at hospital premises in the absence of any providers to minimize such bias.

### Conclusions

Our findings demonstrate that more than half of the patients were overall satisfied with the services provided under the SSK scheme. However, there is room for improvement in several dimensions, such as the cleanliness of the waiting room and toilets and the availability of drinking water. Furthermore, attention should be paid to minimizing the waiting time for registration and accessing health care services, and improving providers’ skills on interaction with patients. The results of this study could help stakeholders make necessary changes in the identified determinants of satisfaction related to health service delivery of the SSK scheme. Such changes will enhance the quality of services as well as increase utilization of the scheme in the target population, ultimately advancing progress toward achieving universal health coverage.

## Appendix

Multimedia Appendix 1 Overall satisfaction with the inpatient care services under the Shasthyo Surokhsha Karmasuchi (SSK) scheme.

## Abbreviations

LMIC: low- and middle-income country
SSK: Shasthyo Surokhsha Karmasuchi
UzHC: Upazila Health Complex

## Data Availability

The data sets generated or analyzed during this study are available from the corresponding author upon reasonable request.
