# Supplementary figures and images for: Patient Satisfaction With the Health Care Services of a Government-Financed Health Protection Scheme in Bangladesh: Cross-Sectional Study
Source: JMIR Form Res. 2024 Apr 24;8:e49815. doi: 10.2196/49815 (PMC11079759; doi:10.2196/49815)

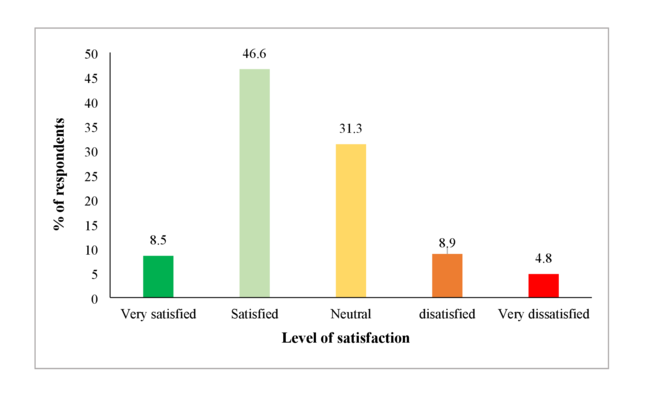

Supplement: Multimedia Appendix 1 [file formative_v8i1e49815_app1.png]
